# The impact of the COVID-19 pandemic on renal cancer care

**DOI:** 10.1007/s00345-024-04925-2

**Published:** 2024-04-13

**Authors:** Hilin Yildirim, Adriaan D. Bins, Corina van den Hurk, R. Jeroen A. van Moorselaar, Martijn G. H. van Oijen, Axel Bex, Patricia J. Zondervan, Katja K. H. Aben

**Affiliations:** 1 Cancer Center Amsterdam, Amsterdam UMC, University of Amsterdam, 4F, De Boelelaan 1117, 1081 HV Amsterdam, The Netherlands; 2 Department of Research and Development, Netherlands Comprehensive Cancer Organisation, Utrecht, The Netherlands; 3 Department of Medical Oncology, Amsterdam UMC, University of Amsterdam, Amsterdam, The Netherlands; 4 Department of Urology, Amsterdam UMC, Vrije Universiteit Amsterdam, Amsterdam, The Netherlands; 5 Department of Urology, The Netherlands Cancer Institute, Antoni van Leeuwenhoek Hospital, Amsterdam, The Netherlands; 6 The Royal Free London NHS Foundation Trust, London, UK; 7 Division of Surgery and Interventional Science, University College London, London, UK; 8 Department of Urology, Amsterdam UMC, University of Amsterdam, Amsterdam, The Netherlands; 9 Department for Health Evidence, Radboud University Medical Centre, Nijmegen, The Netherlands

**Keywords:** COVID-19, Renal cell carcinoma, Incidence, Disease stage, Treatment

## Abstract

**Purpose:**

To evaluate the impact of the COVID-19 pandemic on renal cell carcinoma (RCC) care in the Netherlands.

**Methods:**

Newly diagnosed RCCs between 2018 and 2021 were selected from the Netherlands Cancer Registry; 2020–2021 was defined as COVID period and 2018–2019 as reference period. Numbers of RCCs were evaluated using 3-week-moving averages, overall and by disease stage and age. Changes in treatment were evaluated with logistic regression analyses. To evaluate possible delays in care, time to start of treatment was assessed. The cumulative number of metastatic RCC (mRCC) over time was assessed to evaluate stage shift.

**Results:**

During the 1st COVID wave (weeks 9–22, 2020), the number of new RCC diagnoses decreased with 15%. Numbers restored partially in 2020, but remained 10% lower compared to 2018/2019. The decline was mostly due to a drop in T1a/T1b RCCs and in age > 70 years. 2021 showed similar numbers of new RCC diagnoses compared to 2018/2019 without an increase due to previously missed RCCs. Treatment-related changes during the 1st COVID wave were limited and temporarily; less surgery in T1a RCCs in favor of more active surveillance, and in mRCC targeted therapy was preferred over immunotherapy. Time to start of firstline treatment was not prolonged during the 1st COVID wave. No increase in mRCC was found until the end of 2021.

**Conclusions:**

The COVID-19 pandemic resulted in fewer RCC diagnoses, especially T1a/T1b tumors. Treatment-related changes appeared to be limited, temporarily and in accordance with the adapted guidelines. The diagnostic delay could lead to more advanced RCCs in later years but there are no indications for this yet.

**Supplementary Information:**

The online version contains supplementary material available at 10.1007/s00345-024-04925-2.

## Introduction

The COVID-19 pandemic was caused by a novel coronavirus (Severe Acute Respiratory Syndrome–coronavirus-2, or SARS–CoV-2) and put a strain on healthcare. The first COVID-19 positive patient in the Netherlands was diagnosed on February 27th 2020 [1]. Evolving in the Southern part of the country, the virus spread gradually to the rest of the country. To prevent further spreading and overload in hospitals, a national lockdown was announced on March 23rd 2020. The increased number of hospitalized patients with a COVID-19 infection led to downscaling of medical care. All none-urgent appointments, procedures and treatments were postponed or cancelled. At the same time, patients who feared becoming infected or did not want to burden the healthcare system, avoided (urgent) medical care [2]. As a result, during the first COVID-19 wave a significant decrease of 25% in cancer diagnoses was reported in the Netherlands [2, 3].

Adapted (inter)national guidelines were published to guide downscaling of regular care [4–7]. Specifically for renal cancers, the Dutch Urological Association (NVU) prioritized surgical treatments based on their urgency. Partial nephrectomies and focal therapies were considered less urgent and it was recommended to perform these procedures only if surgical capacity was available. The recommendation in Dutch guidelines for radical nephrectomies was not changed; perform surgery within 6 weeks [7]. The Dutch Association of Medical Oncology (NVMO) recommended to delay systemic therapy (in metastatic disease) if possible. Specifically for renal cancer, it was advised to cancel maintenance immunotherapy and to consider replacement of immunotherapy with targeted therapy (tyrosine kinase inhibitors) [6].

The impact of the COVID-19 pandemic and the subsequent downscaling of regular healthcare in the Netherlands on renal cancer care is largely unknown. Therefore, we aimed to evaluate this impact on the number of new renal cell carcinoma (RCC) diagnoses, age and disease stage and treatment. In addition, the effect on surgical capacity in hospitals was evaluated.

## Materials and methods

All patients newly diagnosed with renal cancer between January 2018 and December 2021 were identified through the population-based Netherlands Cancer Registry (NCR) and included in this historic cohort study [8]. Data on patient, tumor and treatment characteristics were extracted from the NCR. Detailed description of the variables and used definitions are described in Appendix A.

Patients diagnosed in 2020 and 2021 were considered as the COVID cohort and patients diagnosed in 2018/2019 as the reference cohort. As the impact of the COVID-19 outbreak in general and specifically on clinical care was most prominent in 2020, we divided the year 2020 into four distinct time periods based on COVID-19-related public restrictions: pre-COVID (weeks 1–8, 2020), 1st COVID wave (weeks 9–22, 2020; in week 9 the first COVID-19 patient was diagnosed in the Netherlands and in week 13 the first national lockdown started which ended in week 22), 2nd COVID period without lockdown (weeks 23–40 in 2020), 3rd COVID period with (partial) lockdown (weeks 41–52, 2020; the Netherlands experienced a period of different restrictions and (partial) lockdowns).

Descriptive analyses were performed to provide insight in the COVID cohort and the reference cohort. Three-week-moving averages were used to evaluate the number of new diagnoses in the COVID period vs. the reference period. Due to small numbers, a 3-week moving average was used to evaluate trends over time, smoothing the average number per time period. In addition, the relative change in the number of diagnoses in 2020 and 2021 was evaluated by considering 2018/2019 as 100%. Logistic regression analyses were performed to evaluate age-adjusted probability of receiving a certain treatment and Mann–Whitney *U* tests were used to compare time since diagnosis to start treatment. A more detailed description of the statistical analyses is given in Appendix A. All statistical analyses were performed using SAS version 9.4 (SAS Institute, Cary, North Caroline, USA) and STATA version 16.1 software (StataCorp, College Station, Texas, USA). *p* value < 0.05 was considered as statistically significant.

## Results

### New RCC diagnoses

Patient- and tumor characteristics of the COVID cohort (divided in distinct periods) and the reference cohort were described in Table 1.

**Table 1:** Baseline characteristics of patients diagnosed with renal cancer in 2020 (divided in time periods), in 2021, and in the reference period 2018/2019

	2018–2019	Pre-COVID: week 1–8 2020	1st COVID wave: week 9–22 2020	2nd COVID period without lockdown: week 23–40 2020	3rd COVID period with (partial) lockdown: week 41–52 2020	2021
Total number of patients, *n* (%)	5665 (100%)	407 (100%)	639 (100%)	862 (100%)	655 (100%)	2797 (100%)
Gender, *n* (%)						
Male	3719 (65.6)	279 (68.6%)	426 (66.7%)	565 (65.5%)	421 (64.3%)	1797 (64.2%)
Female	1946 (34.4)	128 (31.4%)	213 (33.3%)	297 (34.5%)	234 (35.7%)	1000 (35.8%)
Age at diagnosis						
Median (IQR)	69.0 (60.0–75.0)	68.0 (59.0–75.0)	67.0 (59.0–75.0)	68.5 (60.0–76.0)	70.0 (62.0–75.0)	68.0 (59.0–76.0)
Mean (SD)	67.3 (11.9)	67.1 (11.9)	66.8 (11.4)	67.5 (11.6)	68.0 (11.3)	67.3 (11.7)
Age at diagnosis, *n* (%)						
< 60	1404 (24.8%)	107 (26.3%)	163 (25.5%)	206 (23.9%)	140 (21.4%)	714 (25.5%)
61–70	1563 (27.6%)	114 (28.0%)	197 (30.8%)	246 (28.5%)	186 (28.4%)	748 (26.7%)
71–80	1850 (32.7%)	135 (33.2%)	187 (29.3%)	284 (32.9%)	243 (37.1%)	948 (33.9%)
81 +	848 (15.0%)	51 (12.5%)	92 (14.4%)	126 (14.6%)	86 (13.1%)	387 (13.8%)
Clinical tumor size at diagnosis (mm)						
Median (IQR)	50.0 (30.0–79.0)	48.0 (30.0–80.0)	50.0 (32.0–81.0)	50.0 (30.0–81.0)	51.0 (32.0–80.0)	50.0 (30.0–80.0)
Mean (SD)	58.0 (36.4*)*	57.1 *(35.2)*	59.8 (37.0)	58.6 (36.5)	59.0 (35.6)	58.2 (36.8)
M stage, *n* (%)						
M0	4656 (82.2%)	327 (80.3%)	516 (80.8%)	673 (78.1%)	532 (81.2%)	2272 (81.2%)
M1	1009 (17.8%)	80 (19.7%)	123 (19.2%)	189 (21.9%)	122 (18.6%)	520 (18.6%)
Unknown	0 (0.0%)	0 (0.0%)	0 (0.0%)	0 (0.0%)	1 (0.2%)	5 (0.2%)
Disease stage (TNM), *n* (%)						
Stage 1	3309 (58.4%)	235 (57.7%)	348 (54.5%)	485 (56.3%)	369 (56.3%)	1612 (57.6%)
Stage 2	691 (12.2%)	51 (12.5%)	83 (13.0%)	101 (11.7%)	86 (13.1%)	327 (11.7%)
Stage 3	330 (5.8%)	21 (5.2%)	51 (8.0%)	47 (5.5%)	38 (5.8%)	166 (5.9%)
Stage 4	1236 (21.8%)	96 (23.6%)	145 (22.7%)	219 (25.4%)	150 (22.9%)	649 (23.2%)
Unknown	99 (1.7%)	4 (1.0%)	12 (1.9%)	10 (1.2%)	12 (1.8%)	43 (1.5%)
Morphology, *n* (%)						
No histological confirmation	1156 (20.4%)	79 (19.4%)	119 (18.6%)	191 (22.2%)	129 (19.7%)	562 (20.1%)
Clear cell	2958 (52.2%)	195 (47.9%)	328 (51.3%)	442 (51.3%)	358 (54.7%)	1406 (50.3%)
Papillary	592 (10.5%)	49 (12.0%)	65 (10.2%)	83 (9.6%)	72 (11.0%)	313 (11.2%)
Chromophobe	232 (4.1%)	18 (4.4%)	30 (4.7%)	29 (3.4%)	26 (4.0%)	121 (4.3%)
Sarcomatoid	58 (1.0%)	1 (0.2%)	5 (0.8%)	3 (0.3%)	1 (0.2%)	11 (0.4%)
RCC NOS	511 (9.0%)	53 (13.0%)	77 (12.1%)	88 (10.2%)	57 (8.7%)	282 (10.1%)
Other	158 (2.8%)	12 (2.9%)	15 (2.3%)	26 (3.0%)	12 (1.8%)	102 (3.6%)

**Fig. 1:**
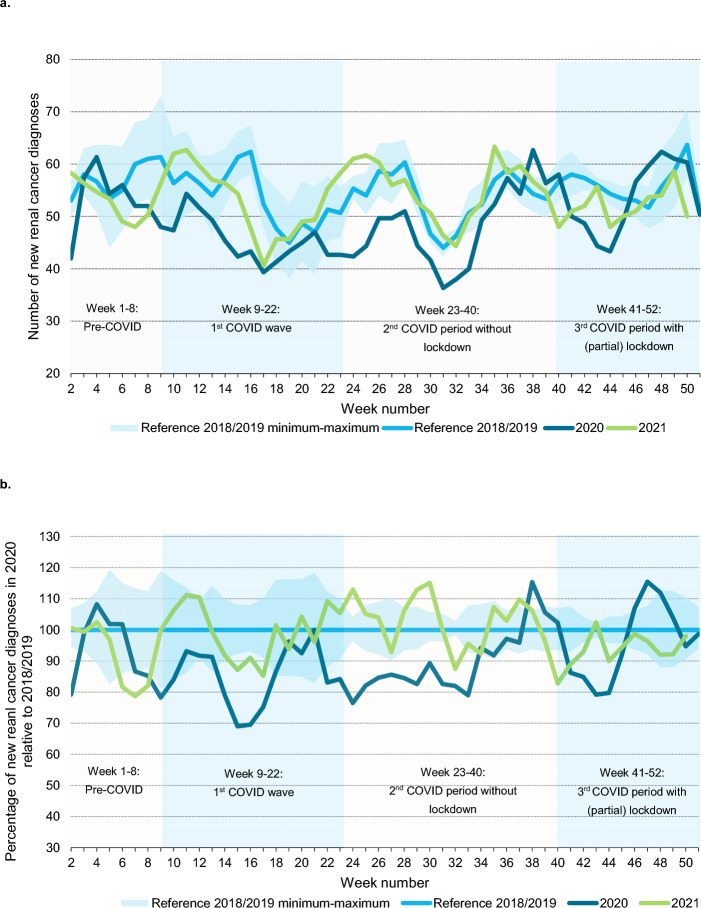
Three week moving averages of newly diagnosed renal cancers (absolute numbers) in the Netherlands in 2020 and 2021 compared to the reference period 2018/2019 (**a**) and relative to the reference period 2018/2019 (**b**)

In Fig. 1(a, b) 3-week moving averages of new RCC diagnoses are presented for 2020 and 2021 with 2018/2019 as reference period. During the 1st COVID wave, an initial decline of 30% in RCC diagnoses was found, followed by decreased numbers up to 20% in subsequent periods. After week 38, short periods with increased numbers (up to 15%) were observed. Overall, the total number of RCC diagnoses in 2020 remained 10% (*N* ~ 270) lower than expected based on 2018/2019. In 2021 the number of new RCC diagnoses was comparable to 2018/2019.

### Disease stage and age at diagnosis

Figure 2a (supplementary) shows that the observed decline in number of diagnoses in 2020 was largely due to a decline in T1a/T1b tumors (*N* ~ 218); during the 1st COVID wave and the 2nd COVID period without lockdown, numbers were significantly decreased. In the 3rd COVID wave with (partial) lockdown, the number of new T1a/T1b tumors was still slightly lower, but this decline was not statistically significant. In other disease stages a small decline in numbers was observed as well, but altogether not statistically significant. In 2021 no clear differences in the number of diagnoses per disease stage were found (Supplementary Fig. 3a).

In Fig. 2b (supplementary), the incidence of RCC in 2020 was presented by age group; the decline in number of diagnoses was most prominent in elderly patients (> 70 years) during the 1st COVID wave and the 2nd COVID period without lockdown. From week 41 in 2020 onwards, the incidence was not statistically different to the reference years. In 2021 no significant differences in the number of diagnoses by age were found (Supplementary Fig. 3b).

### Treatment

Patients with T1a RCC had more often no active treatment during the 2nd COVID period compared to 2018/2019 (36.2% vs. 30.8%, OR 1.36, 95% CI 1.03–1.80) and less often underwent radical nephrectomy (9.6% vs. 13.8%, OR 0.67, 95% CI 0.45–0.99) (Supplementary Fig. 4 and Supplementary Table 2). In 2021, more often “Other treatment” was applied compared to the reference period. Patients with “Other treatment” mostly had radiotherapy. This increased use of radiotherapy is unlikely to be COVID-related but might reflect a new development in clinical practice for old and frail patients in the Netherlands. No other significant changes in treatment of T1a RCC were observed.

For T1b RCC no statistically significant differences were found in treatment per time period, except for “Other treatment”, similar to the observation in T1a tumors. Also for T2/T3 RCC no statistically significant differences were observed.

Patients with T4 RCC and/or nodal involvement and/or metastatic disease received significantly more often immunotherapy and less often targeted therapy in all time periods of 2020 and in 2021 (only the OR for targeted therapy during the 1st COVID wave in 2020 was not statistically significant) compared to the reference period. The increased use of immunotherapy is probably not COVID-related as this was already seen pre-COVID. The temporary decline in use of immunotherapy and increased use of targeted therapy during the 1st COVID wave in 2020 reflects the recommendation to consider replacement of immunotherapy with targeted therapy (immunotherapy 29.0% in the 1st COVID wave vs. 31.3–33.3% in later periods and targeted therapy 20.7% in the 1st COVID wave vs. 11.4–15.6% in later periods).

### Time to treatment

The median time from diagnosis to (partial) nephrectomy was on average 48 days in 2018/2019 and did not increase during the 1st COVID wave (46 days) and even decreased significantly in later periods (40–43 days, *p* < 0.01). Concerning the start of first-line systemic therapy, median time from diagnosis to the start of first-line systemic therapy (significantly) increased from the 2nd COVID period onwards from 25 to 30–31 days (Supplementary Fig. 5).

### Surgical volume

During the 1st COVID wave, an increase up to approximately 40% more (partial) nephrectomies per week was observed (Supplementary Fig. 6). This increase was followed by a similar decrease during the 2nd COVID period without lockdown. However, overall a decline of 11% in (partial) nephrectomies was observed in 2020 (~ 1513 in 2020 and ~ 1694 in 2018/2019). In 2021 the number of (partial) nephrectomies were more in line with 2018/2019 (~ 1603 in 2021).

### Stage shift over time

The cumulative absolute number of patients with metastatic disease in 2020 and 2021 is presented in Fig. 7 (supplementary) to evaluate early effects of a delayed diagnosis. From May/June onwards in 2020 a small increase in the number of metastatic RCC diagnoses was observed, which falls within the expected range of the reference period. In 2020 and 2021 approximately 520 patients were diagnosed annually with metastatic RCC compared to approximately 500 in 2018–2019.

## Discussion

The current study revealed that the outbreak of COVID-19 in the Netherlands caused an initial decline of 30% in the number of RCC diagnoses. Numbers restored partially, but remained 10% lower in 2020 as compared to 2018/2019. In 2021 numbers were largely similar to those of 2018/2019, but no subsequent increase in RCC diagnoses due to delayed diagnosis was observed. The observed decline was largely due to a decrease in T1a/T1b tumors and most pronounced among elderly. Up to 2021, no evidence of a stage shift towards more advanced RCC due to a diagnostic delay was found. Treatment-related changes during the 1st COVID wave were limited and temporarily, and in adherence to adjusted guidelines [4, 5].

Our analyses showed that the decrease in RCC diagnoses was most pronounced in older people, which is consistent with previous studies evaluating the impact of the COVID-19 pandemic on cancer care [9–11]. Especially, elderly have avoided healthcare services due to their increased vulnerability to a COVID-19 infection, which can be more severe and fatal in this group [12]. The higher mortality rate associated with COVID-19 also might have led to a reduced number of RCC diagnoses, especially in older patients [12]. However, the impact of excess mortality due to COVID-19 on the number of bladder- and prostate cancer was minimal, estimating approximately 15 fewer cases of bladder- and 25 fewer cases of prostate cancer [9, 10]. Therefore, we expect that the impact of the excess mortality on the number of RCC diagnoses is negligible.

As stated before, the decline in RCC diagnoses was mostly due to a drop in T1a/T1b tumors. It is known that the majority of small renal cancers are diagnosed incidentally on imaging modalities [13]. The downscaling of regular medical care and subsequent use of imaging during the COVID-19 wave might explain this observation [14, 15]. Interestingly, no subsequent increase in diagnoses was seen; the number of diagnoses in 2021 was not higher than expected. In addition, no increase in more advanced stage RCCs was found. These results are in line with the results of a small cohort study from Italy. They reported a decrease of 10% in the number of RCC diagnoses in 2020 compared to 2018–2019 (*N* ~ 91 vs. 101), without evidence of a short-term trend towards advanced stage tumors in 2020 [16]. Our hypothesis is that part of the patients who formerly had an incidentally detected renal tumor did not seek medical attention once their complaints/symptoms became self-limiting and they no longer required a visit to a health-care provider. On short term, it is not expected that this would lead to more advanced cancers, as small renal tumors have a slow growth rate [17]. However, on long term, delayed presentation of undiagnosed renal cancers could possibly lead to advanced stages and potentially impact survival [18].

Next to the impact of the COVID-19 pandemic on RCC diagnoses, we evaluated treatment-related changes. T1a RCC was more often managed with active surveillance and less often with surgery during the 2nd COVID period without lockdown in 2020. This might be explained by hospitals focusing on (catching up of) urgent (oncological) surgeries and T1a RCC can be managed with active surveillance postponing surgery.

Following adapted treatment guidelines, less patients with T4 and/or N + and/or M + disease received immunotherapy during the 1st COVID wave and targeted therapy was applied more often. This observation is consistent with the outcome of an international online survey among physicians involved in the treatment of metastatic RCC who preferred targeted therapy over immunotherapy regimens, whereas prior to the pandemic, ipilimumab/nivolumab was the preferred choice in intermediate/poor risk patients [19]. The temporarily decreased use of immunotherapy might affect survival rates of patients with advanced RCC since long-term survival is improved in patients treated with first-line combination immunotherapy than those treated with sunitinib [19–21].

Time to first-line systemic therapy was slightly prolonged following the 1st COVID wave. For patients with metastatic RCC, systemic therapy is initiated based on risk stratification, radiological progression, and/or symptomatic disease. Delaying systemic therapy for metastatic RCC has shown to have little impact on overall survival, especially when there are limited metastases. Nevertheless, an optimal approach to select these patients has not yet been established [22].

After the COVID-19 outbreak, time to (partial) nephrectomy was not increased during the 1st COVID wave and even became shorter in the subsequent periods. In anticipation of a potential worsening of the COVID-19 situation, hospitals might have fully addressed waiting lists for oncological purposes.

We observed that initially (during the 1st COVID wave) the number of nephrectomies was increased compared to what was expected based on the reference period. This is in line with findings from other studies [9, 10, 23]. A explanation might be that despite the reduced surgical capacity of hospitals during the 1st COVID wave, oncological surgeries were prioritized. In 2020, however, the total number of surgeries was slightly lower compared to previous years which might be the result of fewer diagnoses and the preference for other nephron sparing managements such as active surveillance and focal therapy, particularly for T1a/T1b RCC [24–26].

Although our study is based on a large nationwide registry, there are some limitations to be considered. Despite the use of nationwide data, some subgroup analyses were based on small numbers. In addition, the reference period was defined as 2018/2019 and trends over time were not taken into account. However, we assume that this effect is minimal since the incidence of RCC was stabilizing in recent years in the Netherlands. A slight underestimation of undiagnosed RCC cases cannot be excluded [27].

## Conclusions

Overall, during the 1st COVID wave health care providers were able to provide RCC care in accordance with the adapted treatment guidelines in the Netherlands. A decline of approximately 10% in all RCC diagnoses was observed in 2020, mostly in T1a/T1b tumors and among elderly. No increase of RCC diagnoses in 2021 was found due to the decline in 2020. There was no evidence for more advanced stage disease until 2021. However, long-term consequences of a potential stage shift due to a diagnostic delay and its impact on the oncological outcomes should be assessed in future research.

## Supplementary Information

Below is the link to the electronic supplementary material.

Supplementary file1 (DOCX 38 KB)

Supplementary file2 (PDF 42 KB)

Supplementary file3 (PDF 198 KB)

Supplementary file4 (PDF 330 KB)

Supplementary file5 (PDF 181 KB)

Supplementary file6 (PDF 13 KB)

Supplementary file7 (PDF 8 KB)

Supplementary file8 (PDF 65 KB)

## Data Availability

All data from the Netherlands Cancer Registry are available on reasonable request (https://www.iknl.nl/en/ncr/apply-for-data).
