# Supplementary material for: The impact of the COVID-19 pandemic on renal cancer care
Source: World J Urol. 2024 Apr 13;42(1):231. doi: 10.1007/s00345-024-04925-2 (PMC11016011; doi:10.1007/s00345-024-04925-2)
Supplement: Supplementary file 1 — Supplementary file1 (DOCX 38 KB) [file 345_2024_4925_MOESM1_ESM.docx]

**Appendix A**

*Definitions*

The NCR receives notifications of new cancer diagnoses from the automated nationwide network and registry of histo- and cytopathology (PALGA). In addition, renal cancers without pathological confirmation were retrieved by a linkage of the Dutch Hospital Data (DHD) to the NCR. Detailed data on patient characteristics (gender, age at diagnosis, postal code), tumor characteristics (morphology according to the international classification for Oncology (ICD-O) third edition [1] and TNM disease stage according to the UICC Tumor-Node Metastasis classification [1,2], and type (and date of start) of first-line treatment were available through the NCR.

Age at diagnosis was evaluated continuously and by category: <60, 60-70, 70-80 and >80 years.

All renal cancers (any grade) were categorized in four stage groups: 1) cT1a/N0-X/M0-X, 2) cT1b/N0-X/M0-X, 2) cT2-T3/N0-X/M0-X) and 4) cT4 and/or N+ and/or M+. Treatment was divided in ‘no active treatment’ (both including active surveillance and watchful waiting as it was not possible to differentiate between both based on the documentation in the medical files), partial nephrectomy, radical nephrectomy, focal therapy, targeted therapy, immunotherapy and other.

*Statistical analyses*

Descriptive statistics were used to give insight into patient- and tumor characteristics of patients diagnosed in 2020 (stratified by different time periods) and in 2021, versus patients diagnosed in the reference period 2018/2019.

The number of newly diagnosed renal cancers in 2020 and 2021 was calculated per week by using three-week moving averages and compared to 2018/2019 (averaged). Additionally, the relative change in the number of diagnoses was evaluated by considering the three-week moving average of 2018/2019 as 100%.

Incidence rates per 100.000 person years were calculated per predefined time period in 2020 and 2021 and compared to (the same period in) 2018/2019, stratified by disease stage and age, using the iri command in STATA.

For each disease stage, the distribution of different first-line treatments was described by time period in 2020 and 2021 versus the reference. Logistic regression analyses were performed to evaluate the age-adjusted probability of receiving a certain treatment in a time period in 2020 and 2021 compared to the reference period. Time from diagnosis to (partial) nephrectomy and systemic therapy (immunotherapy and/or targeted therapy) was calculated, stratified per time period in 2020 and 2021 and compared to the reference period using the Mann-Whitney U test.

To evaluate the effect of the COVID-19 outbreak on surgical volume, the three-week moving averages of the number of partial and radical nephrectomies in 2020 and 2021 were compared to the reference period by considering the reference period as 100%.

Finally, to provide some insight in whether a possible delay in diagnosis affected the disease stage at diagnosis, we assessed the cumulative number of patients diagnosed with metastatic disease per month in 2020 and 2021 and compared this with 2018/2019.

1. Percy C, Henson D, Thomas LB, Graepel P (1977) International classification of diseases for oncology (ICD-O). Pathologist,College of American Pathologists 31:402–403. https://doi.org/10.1016/s0031-3025(16)37827-8

2. Fritz A, Percy C, Jack A, et al (2000) International classification of diseases for oncology, (ICD-O-3), 3rd editio. World Health Organization
