# Supplementary material for: The impact of the COVID-19 pandemic on renal cancer care
Source: World J Urol. 2024 Apr 13;42(1):231. doi: 10.1007/s00345-024-04925-2 (PMC11016011; doi:10.1007/s00345-024-04925-2)
Supplement: Supplementary file 2 — Supplementary file2 (PDF 42 KB) [file 345_2024_4925_MOESM2_ESM.pdf]

Figure 2. Incidence of renal cancer per 100.000 person years per disease stage (a) and age at diagnosis (b) per time period in 2020 compared to the same time period in 2018/2019.

a.

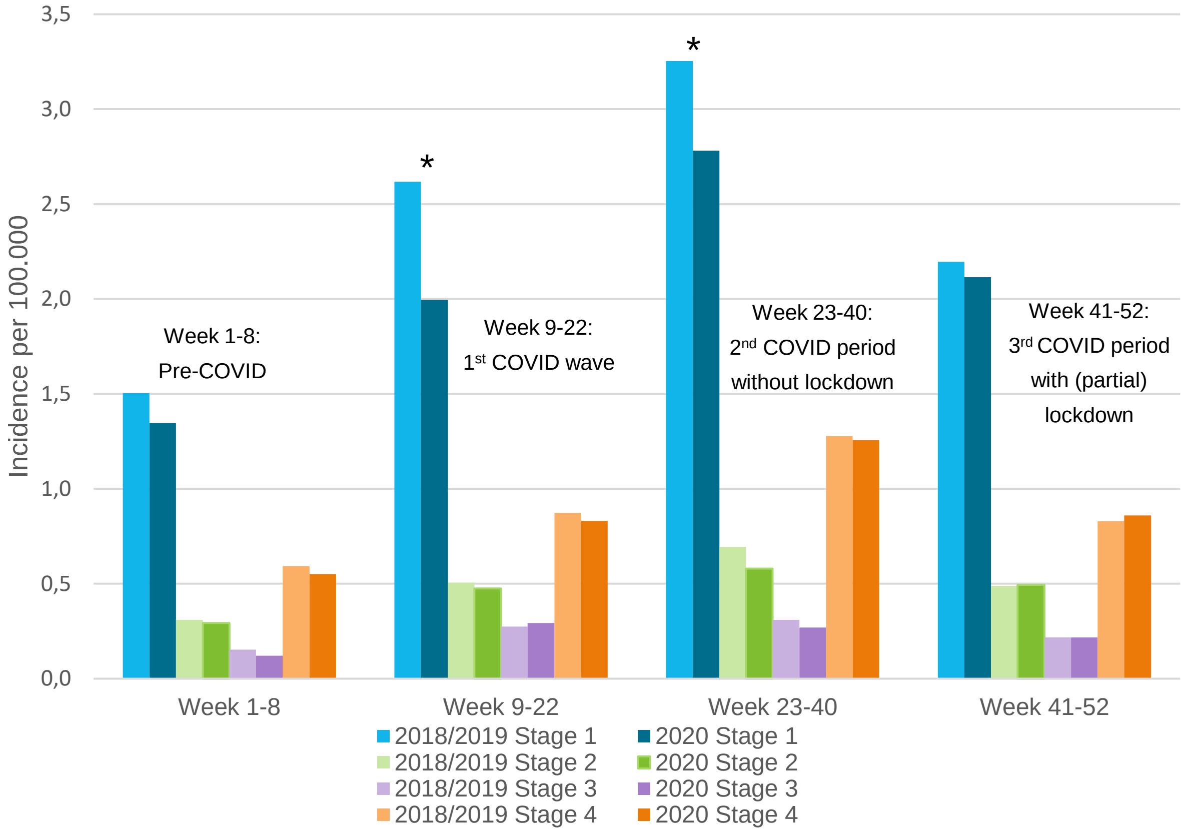

* The incidence is significantly lower (p<0.01) compared to the incidence of 2018/2019.

b.

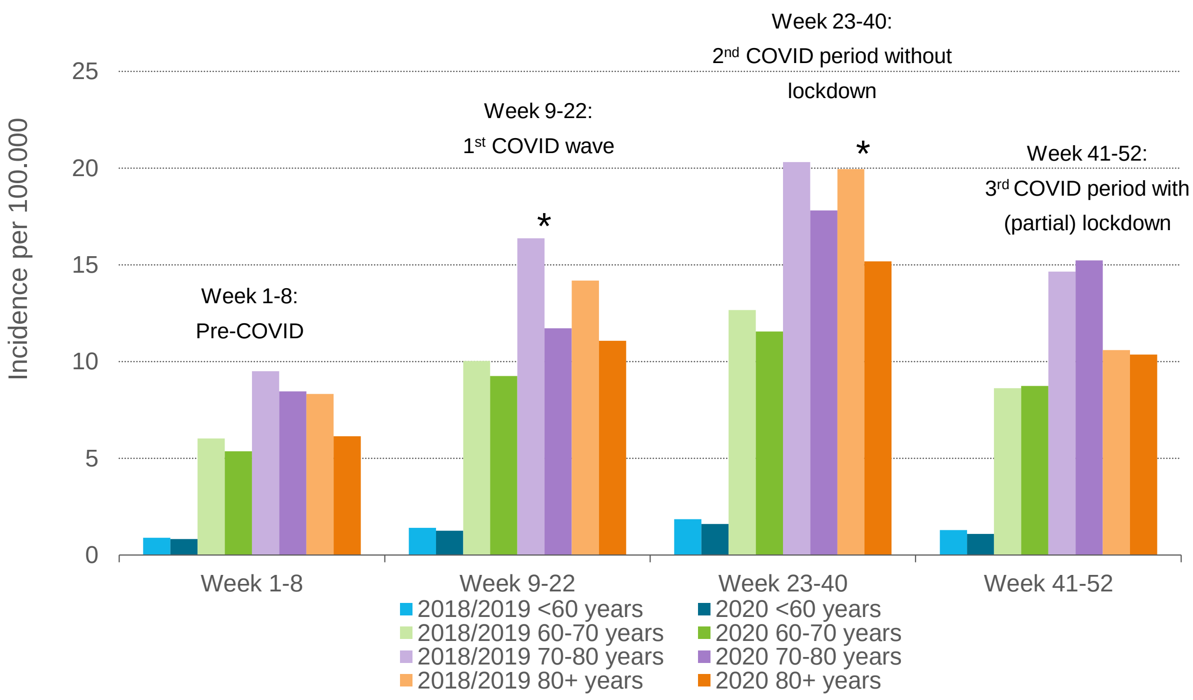

* The incidence is significantly lower (p<0.01) compared to the incidence of 2018/2019.
