# Supplementary material for: The impact of the COVID-19 pandemic on renal cancer care
Source: World J Urol. 2024 Apr 13;42(1):231. doi: 10.1007/s00345-024-04925-2 (PMC11016011; doi:10.1007/s00345-024-04925-2)

Figure 3. Incidence of renal cancer per 100.000 person years by (a) disease stage at diagnosis and (b) age at diagnosis in 2021 and 2018/2019.

a.

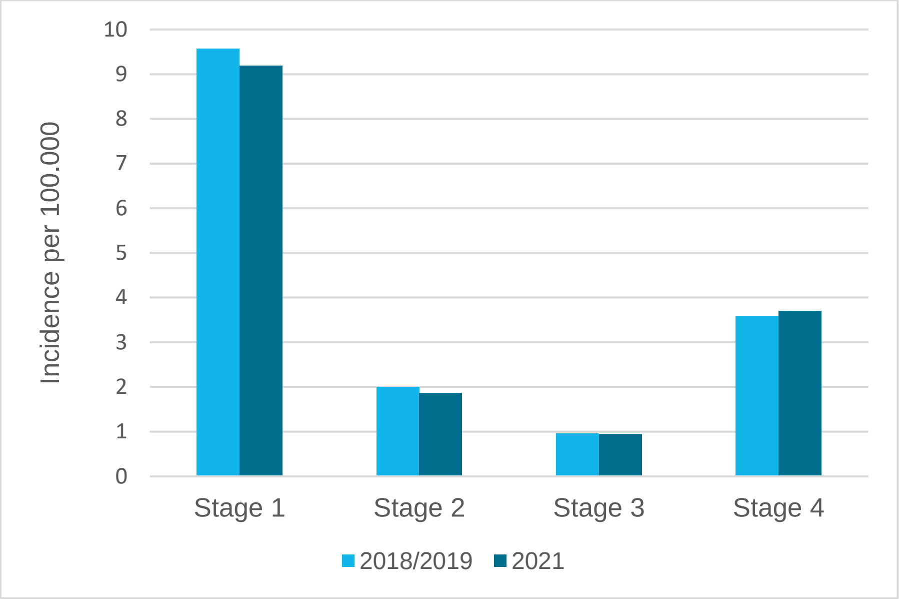

b.

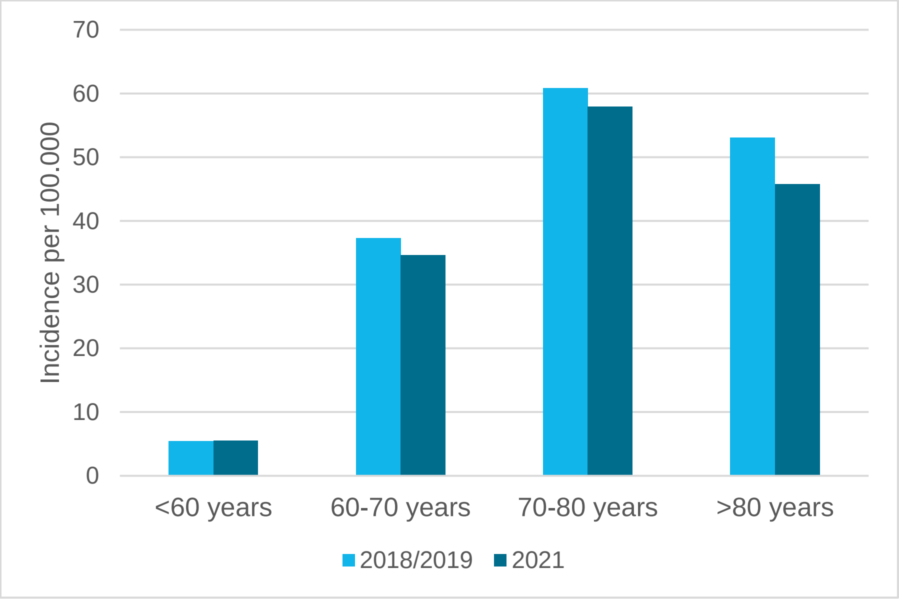

Supplement: Supplementary file 3 — Supplementary file3 (PDF 198 KB) [file 345_2024_4925_MOESM3_ESM.pdf]
