# Supplementary material for: The impact of the COVID-19 pandemic on renal cancer care
Source: World J Urol. 2024 Apr 13;42(1):231. doi: 10.1007/s00345-024-04925-2 (PMC11016011; doi:10.1007/s00345-024-04925-2)
Supplement: Supplementary file 4 — Supplementary file4 (PDF 330 KB) [file 345_2024_4925_MOESM4_ESM.pdf]

Figure 4. First-line treatment of patients diagnosed with renal cancer per disease stage and per period of diagnosis in 2020 and reference period 2018/2019.

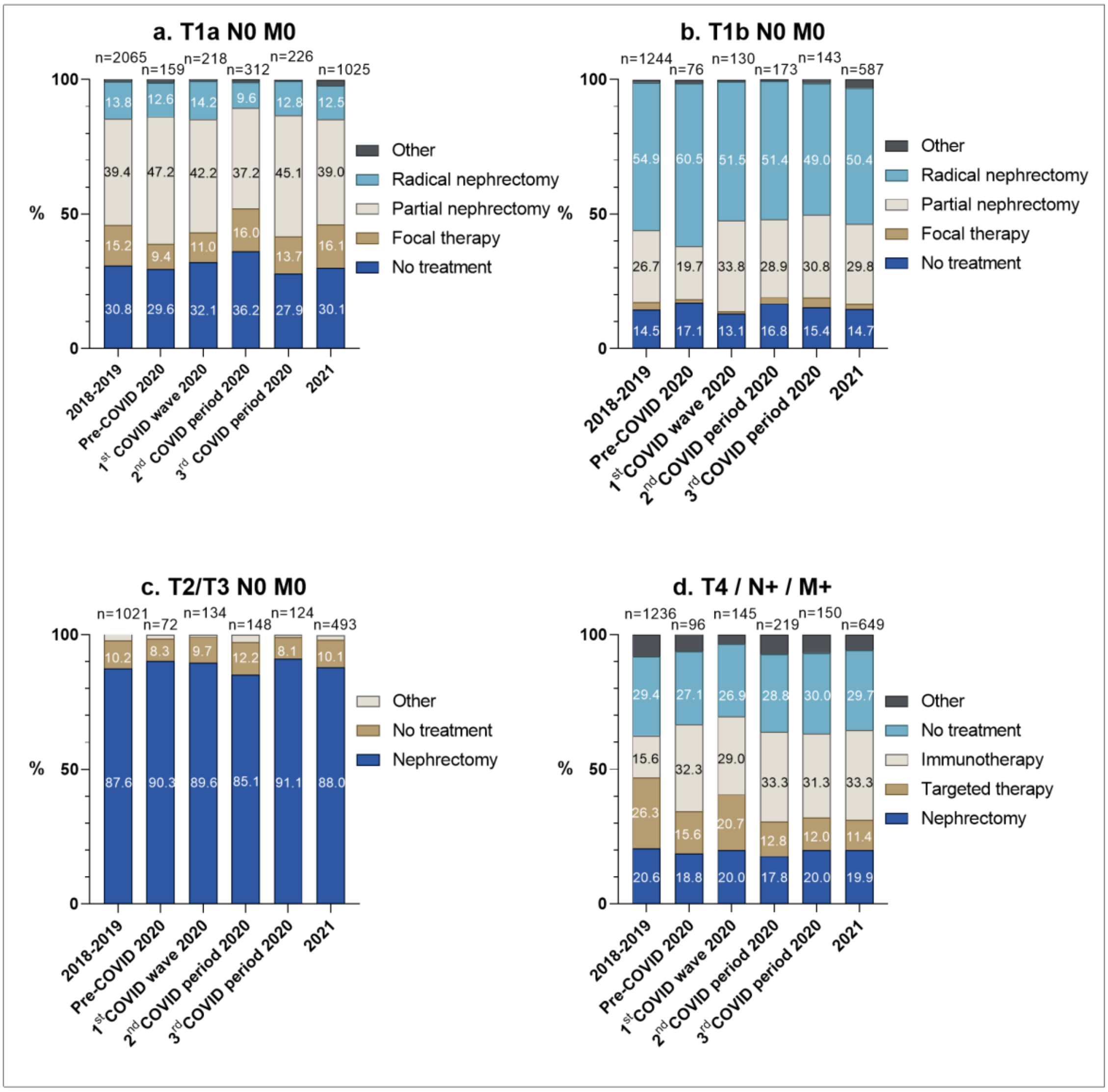

COVID periods in 2020: Pre-COVID: week 1-8 2020, 1st COVID wave: week 9-22 2020, 2nd COVID period without lockdown: week 23-40 2020, 3rd COVID period with (partial) lockdown: week 41-52 2020
