# Supplementary material for: The impact of the COVID-19 pandemic on renal cancer care
Source: World J Urol. 2024 Apr 13;42(1):231. doi: 10.1007/s00345-024-04925-2 (PMC11016011; doi:10.1007/s00345-024-04925-2)
Supplement: Supplementary file 5 — Supplementary file5 (PDF 181 KB) [file 345_2024_4925_MOESM5_ESM.pdf]

Figure 5. Median time with upper and lower quartile (in days) from diagnosis to (a) surgery (partial and radical nephrectomy) and (b) systemic therapy (immuno- and targeted therapy) per period in 2020 and in 2021 compared to the reference period 2018/2019.

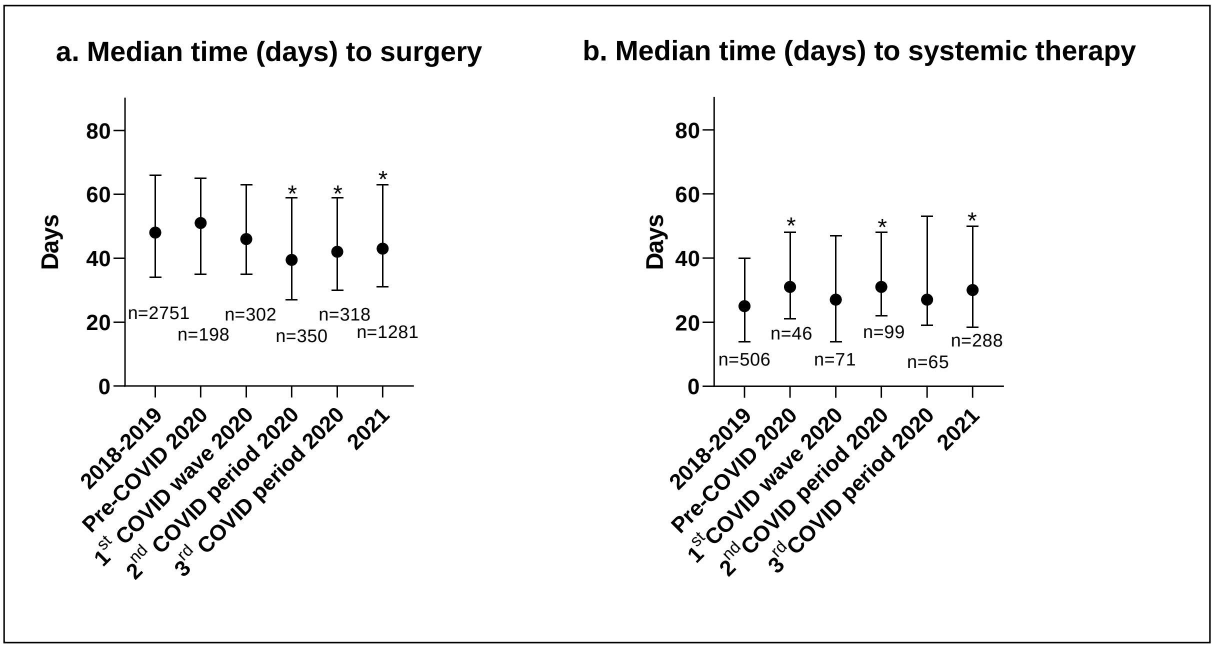

* Time to treatment is significantly lower or higher ($p<0.05$), using the Mann-Whitney U test compared to 2018/2019.
