# Supplementary material for: The impact of the COVID-19 pandemic on renal cancer care
Source: World J Urol. 2024 Apr 13;42(1):231. doi: 10.1007/s00345-024-04925-2 (PMC11016011; doi:10.1007/s00345-024-04925-2)

**Figure 6.** 3-week moving averages of number of (partial) nephrectomies in 2020 and 2021 relative to the reference period 2018/2019 (100%).

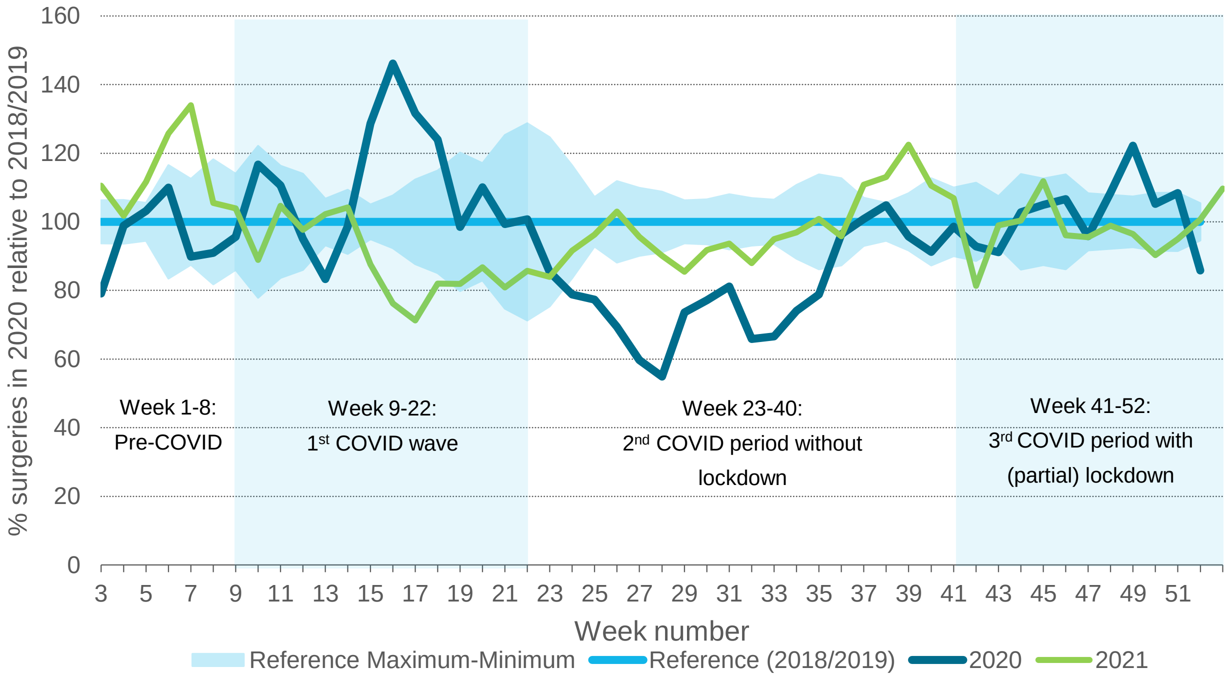

Supplement: Supplementary file 6 — Supplementary file6 (PDF 13 KB) [file 345_2024_4925_MOESM6_ESM.pdf]
