# Supplementary material for: The impact of the COVID-19 pandemic on renal cancer care
Source: World J Urol. 2024 Apr 13;42(1):231. doi: 10.1007/s00345-024-04925-2 (PMC11016011; doi:10.1007/s00345-024-04925-2)

Figure 7. Cumulative number of patients newly diagnosed with metastatic renal cancer in 2020 and 2021 compared to the reference period 2018/2019.

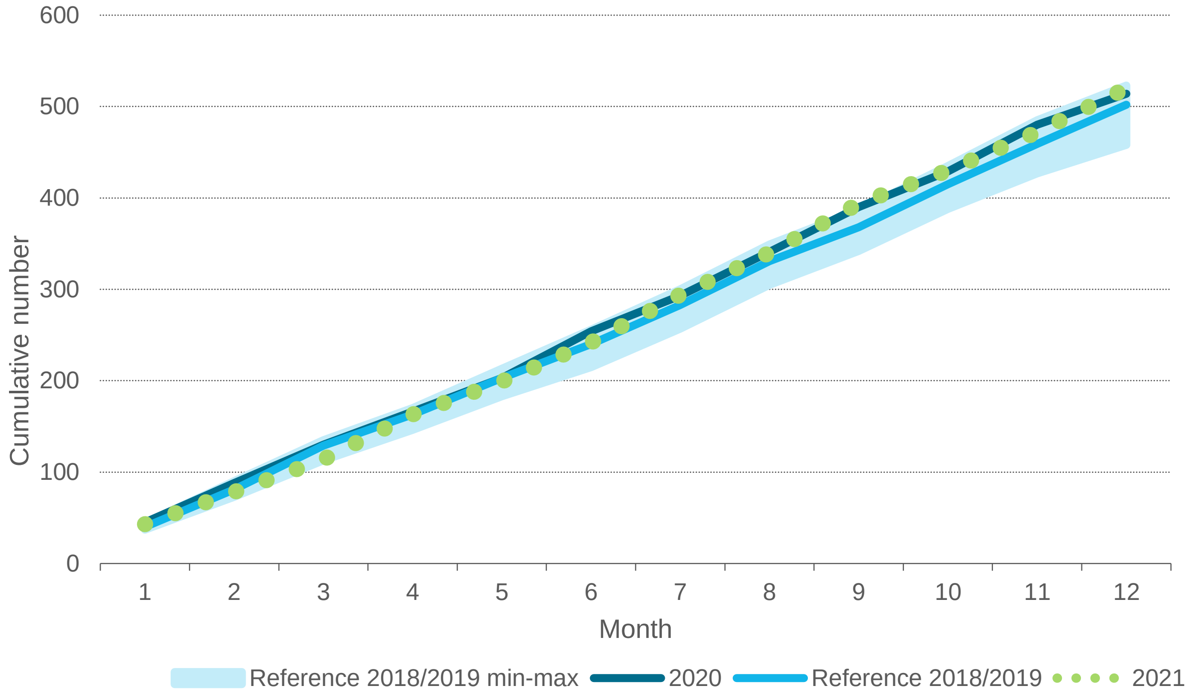

Supplement: Supplementary file 7 — Supplementary file7 (PDF 8 KB) [file 345_2024_4925_MOESM7_ESM.pdf]
