# Supplementary material for: The impact of the COVID-19 pandemic on renal cancer care
Source: World J Urol. 2024 Apr 13;42(1):231. doi: 10.1007/s00345-024-04925-2 (PMC11016011; doi:10.1007/s00345-024-04925-2)
Supplement: Supplementary file 8 — Supplementary file8 (PDF 65 KB) [file 345_2024_4925_MOESM8_ESM.pdf]

Table 2. Logistic regression analyses with Odds Ratios (OR) of receiving treatment per disease stage and per period in 2020 and in 2021 compared to the reference period 2018/2019, adjusted for age at diagnosis.

**T1a N0 M0**	**Reference 2018-2019**		**Pre-COVID Week 1-8 2020**		**1st COVID wave Week 9-22 2020**		**2nd COVID Period without lockdown Week 23-40 2020**		**3rd COVID period with (partial) lockdown Week 41-52 2020**		**2021**	
	OR	95% CI	OR	95% CI	OR	95% CI	OR	95% CI	OR	95% CI	OR	95% CI
No treatment	ref		1.05	0.71-1.57	1.17	0.83-1.64	**1.36**	**1.03-1.80**	0.86	0.61-1.20	0.96	0.80-1.16
Focal therapy	ref		0.59	0.34-1.01	0.69	0.45-1.08	1.07	0.77-1.48	0.89	0.60-1.33	1.07	0.88-1.32
Partial nephrectomy	ref		1.33	0.94-1.88	1.09	0.81-1.48	0.92	0.71-1.20	1.30	0.97-1.75	0.98	0.83-1.15
Radical nephrectomy	ref		0.89	0.55-1.44	1.03	0.69-1.54	**0.67**	**0.45-0.99**	0.92	0.61-1.39	0.90	0.72-1.12
Other	ref		1.47	0.34-6.39	0.53	0.07-3.98	1.10	0.32-3.76	0.51	0.07-3.81	**2.61**	**1.40-4.86**

**T1b N0 M0**	**Reference 2018-2019**		**Pre-COVID Week 1-8 2020**		**1st COVID wave Week 9-22 2020**		**2nd COVID Period without lockdown Week 23-40 2020**		**3rd COVID period with (partial) lockdown Week 41-52 2020**		**2021**	
	OR	95% CI	OR	95% CI	OR	95% CI	OR	95% CI	OR	95% CI	OR	95% CI
No treatment	ref		1.21	0.56-2.24	0.85	0.47-1.54	1.35	0.83-2.19	0.92	0.53-1.58	1.02	0.75-1.40
Focal therapy	ref		0.45	0.06-3.31	0.27	0.04-1.98	0.82	0.29-2.32	1.22	0.47-3.17	0.66	0.33-1.31
Partial Nephrectomy	ref		0.72	0.40-1.29	1.41	0.95-2.09	1.15	0.80-1.64	1.31	0.89-1.93	1.18	0.95-1.48
Radical nephrectomy	ref		1.31	0.81-2.10	0.87	0.60-1.25	0.88	0.64-1.21	0.81	0.57-1.15	0.84	0.69-1.02
Other	ref		1.23	0.16-9.51	0.74	0.10-5.68	0.55	0.07-4.23	1.31	0.29-5.86	**3.17**	**1.55-6.46**

**T2/T3 N0 M0**	**Reference 2018-2019**		**Pre-COVID Week 1-8 2020**		**1st COVID wave Week 9-22 2020**		**2nd COVID Period without lockdown Week 23-40 2020**		**3rd COVID period with (partial) lockdown Week 41-52 2020**		**2021**	
	OR	95% CI	OR	95% CI	OR	95% CI	OR	95% CI	OR	95% CI	OR	95% CI
Nephrectomy	ref		1.49	0.61-3.66	1.29	0.69-2.44	0.88	0.50-1.53	1.44	0.73-2.84	1.07	0.74-1.54
No treatment	ref		0.63	0.26-1.82	0.92	0.47-1.80	1.11	0.61-2.03	0.84	0.41-1.72	0.98	0.66-1.46
Other	ref		0.62	0.08-4.65	0.32	0.04-2.40	1.17	0.40-3.44	0.34	0.05-2.58	0.81	0.37-1.76

**T4 / N1 / M1**	**Reference 2018-2019**		**Pre-COVID Week 1-8 2020**		**1st COVID wave Week 9-22 2020**		**2nd COVID Period without lockdown Week 23-40 2020**		**3rd COVID period with (partial) lockdown Week 41-52 2020**		**2021**	
	OR	95% CI	OR	*95% CI*	OR	95% CI	OR	95% CI	OR	95% CI	OR	95% CI
Nephrectomy	ref		0.89	0.52-1.52	0.90	0.58-1.40	0.82	0.56-1.20	0.97	0.63-1.49	0.94	0.74-1.20
Targeted therapy	ref		**0.52**	**0.29-0.91**	0.70	0.46-1.08	**0.41**	**0.27-0.62**	**0.38**	**0.23-0.63**	**0.36**	**0.27-0.47**
Immunotherapy	ref		**2.73**	**1.71-4.36**	**2.14**	**1.43-3.21**	**2.84**	**2.04-3.96**	**2.62**	**1.78-3.88**	**2.84**	**2.25-3.58**
No treatment	ref		0.95	0.57-1.59	1.00	0.65-1.55	1.00	0.70-1.42	1.00	0.70-1.60	1.05	0.83-1.33
Other	ref		0.77	0.33-1.82	0.42	0.17-1.06	0.91	0.52-1.58	0.81	0.41-1.59	0.69	0.47-1.02

All statistically significant values are in **bold** (P<0.05).

Ref=reference, OR=odds ratio, 95%CI=95% confidence interval
